# Testing mitochondrial sequences and anonymous nuclear markers for phylogeny reconstruction in a rapidly radiating group: molecular systematics of the Delphininae (Cetacea: Odontoceti: Delphinidae)

**DOI:** 10.1186/1471-2148-9-245

**Published:** 2009-10-07

**Authors:** Sarah E Kingston, Lara D Adams, Patricia E Rosel

**Affiliations:** 1 NOAA Fisheries, Southeast Fisheries Science Center, 646 Cajundome Blvd. Suite 234, Lafayette, LA 70506, USA; 2 University of Maryland, College of Chemical and Life Sciences, Program in Behavior, Ecology, Evolution, and Systematics, Biology-Psychology Building 1204C, College Park, MD 20742, USA; 3 Department of Vertebrate Zoology, National Museum of Natural History, Smithsonian Institution, 4210 Silver Hill Rd, Suitland, MD 20746, USA; 4 National Ocean Service, 219 Fort Johnson Road, Charleston, SC 29412, USA

## Abstract

**Background:**

Many molecular phylogenetic analyses rely on DNA sequence data obtained from single or multiple loci, particularly mitochondrial DNA loci. However, phylogenies for taxa that have undergone recent, rapid radiation events often remain unresolved. Alternative methodologies for discerning evolutionary relationships under these conditions are desirable. The dolphin subfamily Delphininae is a group that has likely resulted from a recent and rapid radiation. Despite several efforts, the evolutionary relationships among the species in the subfamily remain unclear.

**Results:**

Here, we compare a phylogeny estimated using mitochondrial DNA (mtDNA) control region sequences to a multi-locus phylogeny inferred from 418 polymorphic genomic markers obtained from amplified fragment length polymorphism (AFLP) analysis. The two sets of phylogenies are largely incongruent, primarily because the mtDNA tree provides very poor resolving power; very few species' nodes in the tree are supported by bootstrap resampling. The AFLP phylogeny is considerably better resolved and more congruent with relationships inferred from morphological data. Both phylogenies support paraphyly for the genera *Stenella *and *Tursiops*. The AFLP data indicate a close relationship between the two spotted dolphin species and recent ancestry between *Stenella clymene *and *S. longirostris*. The placement of the *Lagenodelphis hosei *lineage is ambiguous: phenetic analysis of the AFLP data is consistent with morphological expectations but the phylogenetic analysis is not.

**Conclusion:**

For closely related, recently diverged taxa, a multi-locus genome-wide survey is likely the most comprehensive approach currently available for phylogenetic inference.

## Background

Phylogenetic relationships among cetacean taxa are contended at many different levels. However, robust phylogenies are necessary for gaining insight into the evolutionary histories of these taxa and can help in understanding speciation of highly mobile taxa in an environment with seemingly few barriers to movement. Studies using molecular markers, often mitochondrial DNA (mtDNA) sequences, to elucidate phylogenetic relationships among cetacean taxa have just as often created new controversy as resolved standing controversy [1-8]. At deeper evolutionary levels, nuclear molecular data supported early conclusions based on morphological data that the hippopotamids are the sister lineage to cetaceans within the Artiodactyla [9,10], although, more recent morphological analyses do no support this relationship and instead identify raoellids as the sister group to cetaceans [11]. Within the Cetacea, a previously controversial issue was the placement of sperm whales (Physeteroidea); while initial mtDNA sequence analyses suggested the sperm whale lineage is more closely related to the baleen whales rather than the rest of the toothed whales [4], analysis of mtDNA cytochrome *b *data [12], multiple mtDNA sequences and morphology [13,14] and nuclear data [5,6,15] all support the traditional view of monophyletic suborders. For some cetacean lineages, such as the beaked whales, mitochondrial sequences have proven excellent markers for resolving phylogenies and even pinpointing new species [1,16], although nuclear markers offer new evolutionary insights [17]. For the endangered right whale species, mitochondrial markers render phylogenies congruent with those inferred from nuclear data [18].

One group where relationships among taxa still remain unresolved is the family Delphinidae, and in particular the subfamily Delphininae. This subfamily encompasses eleven nominal species (*sensu *LeDuc *et al*. [3]): *Tursiops truncatus *(Montagu, 1821), *T. aduncus *(Ehrenberg, 1833), *Stenella frontalis *(G. Cuvier, 1829), *S. attenuata *(Gray, 1846), *S. longirostris *(Gray, 1828), *S. clymene *(Gray, 1846), *S. coeruleoalba *(Meyen, 1833), *Delphinus delphis *Linnaeus, 1758, *D. capensis *Gray, 1828, *Lagenodelphis hosei *Fraser, 1956, and *Sousa chinensis *(Osbeck, 1765). Morphological data have most often excluded the genus *Sotalia *Gray, 1866 from this subfamily (but see Kasuya [19]) in concordance with the mtDNA cytochrome *b *phylogeny of LeDuc *et al*. [3], but a recent molecular analysis suggested the genus should be included [20]. However, interpreted in the most conservative manner, the combined analysis of mtDNA and nuclear loci in Caballero *et al*. [20] supports a sister relationship between the lineages leading to the subfamily Delphininae and the genus *Sotalia *but is not necessarily strong evidence for inclusion of *Sotalia *in the subfamily; a subtle but important distinction.

**Figure 1:**
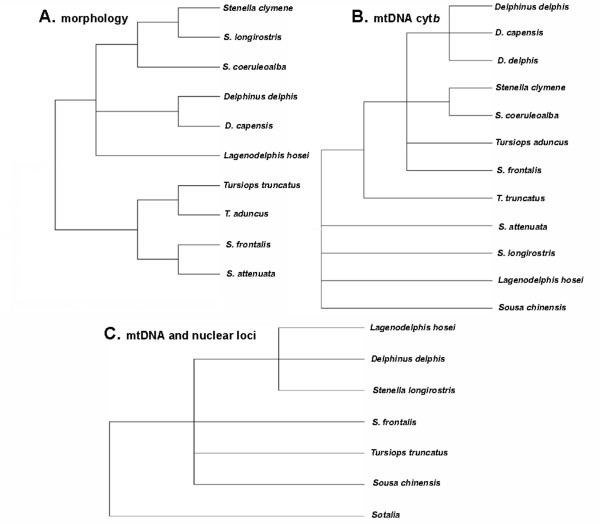
**Hypotheses of the relationships among the nominal species in the subfamily Delphininae**. (*sensu *LeDuc [3]) based on A. recent morphological analyses [26,27,102-104,108,109,115] (none included *Sousa chinensis*), B. full mtDNA cyt*b *sequences [3,21,24,25] in which *Delphinus capensis *was found to be nested within *D*. *delphis*, and C. a combined analysis of 2 mtDNA sequence loci and 10 nuclear gene sequence loci [20] (in which an additional genus, *Sotalia*, is hypothesized to belong to the subfamily).

A number of morphological and genetic studies have been conducted in an attempt to resolve evolutionary relationships among taxa within the family Delphinidae and/or subfamily Delphininae [3,20-27]. LeDuc *et al*. [3] and May-Collado *et al*. [24] used mtDNA cytochrome *b *sequences to reconstruct relationships among the taxa within the family Delphinidae. Although the LeDuc *et al. *[3] phylogeny included representatives of all species within the subfamily Delphininae, each species was represented by only a few sequences (or in the case of May-Collado *et al*. [24], single sequences and an incomplete survey of the taxa within the subfamily); the mitochondrial cytochrome *b *locus was unable to completely resolve branching order within the Delphininae [3,24]. The lack of resolution in the cytochrome *b *phylogeny suggests the Delphininae are the product of a recent radiation; divergence among the numerous taxa is small, rendering resolution of branching order difficult. The LeDuc *et al*. [3] cytochrome *b *phylogeny also suggests polyphyly of the delphinine genera *Stenella *and *Tursiops *and this result for *Stenella *is also supported by May-Collado *et al*. [24]. So far, nuclear and combined analyses concerning the Delphininae raise more questions and provide support for only a few internal nodes within the subfamily [20]. The polyphyletic genera and lack of branching order resolution among many of the delphinine taxa point to a need for a new approach to discerning the evolutionary relationships among the species in this subfamily and perhaps a revision of the subfamily (Figure 1).

We attempted to resolve phylogenetic relationships among members of the subfamily Delphininae using two approaches. Both use complete taxon sampling for the subfamily (*sensu *LeDuc *et al.*[3]) and incorporate multiple individuals per species to capture the intraspecific variation inherent within the species. First, phylogenetic analysis was performed using mitochondrial DNA control region sequences. The control region was chosen for comparison because it is commonly relied upon for studies of cetacean systematics [1,8,22,28,29] and species identification [1,30,31]. The higher mutation rate may allow the control region locus to resolve relationships that the cytochrome *b *gene sequences could not [3,21,24,25]. However, mtDNA sequences represent a single locus gene tree and phylogenetic reconstruction of species trees can be greatly improved through multi-locus analyses. To address this problem, we employed an alternate approach to phylogenetic reconstruction targeting multiple polymorphic markers from anonymous sites across the genome.

Amplified fragment length polymorphism (AFLP) analysis is a powerful molecular technique combining a restriction fragment length polymorphism (RFLP) assay and DNA amplification via the polymerase chain reaction (PCR) [32]. Hundreds of genomic markers can be generated from the assay's restriction enzyme digest and two rounds of fragment amplification via PCR [32,33]. The AFLP method provides a multi-locus approach and may overcome problems in phylogenetic reconstruction resulting from incomplete lineage sorting [34-37]. AFLPs have proven successful for resolving phylogenetic histories at both shallow [38,39] and deeper species level hierarchies [34,40,41]. Within delphinids, these markers are powerful enough to reveal differentiation between two sets of closely related taxa, *Delphinus delphis *and *D. capensis*, and offshore and coastal morphotypes of *T. truncatus *[42]. In addition, Koopman [43] demonstrated substantial phylogenetic signal in AFLP data and congruence between the AFLP markers and other nuclear data (ITS sequences). Here we compare and contrast the efficacy of mtDNA sequences and AFLP markers for reconstructing evolutionary histories in a group that has undergone a rapid radiation.

## Methods

### Sampling - mtDNA

A total of 346 control region mtDNA haplotypes (i.e., unique sequences) representing eleven delphinine species and seven outgroup taxa were utilized for this analysis. Fourteen of the fifteen outgroup taxa haplotypes were downloaded from GenBank as well as 23 *Tursiops aduncus* haplotypes, 7 south Australian *T. truncatus* haplotypes, and one Indopacific *T. truncatus* haplotype (Table 1). The remaining haplotypes resulted from sequences obtained in our lab from 1808 individual samples. Six hundred and six of these samples were sequenced for previous studies: 312 *Delphinus *spp. samples, 199 *S. frontalis *samples, and 95 *Tursiops truncatus *samples [29,42,44-48].

**Table 1:** GenBank accession numbers and species information for samples incorporated to augment species coverage for mtDNA control region analysis.

**GenBank accession #**	**species**	**Reference**
AB018584	*Grampus griseus*	(Yamagiwa, 1998)
AF113486	*Lagenorhynchus acutus*	[116]
AF113490-AF113491	*Lagenorhynchus obliquidens*	[116]
AF113492-AF113496	*Lagenorhynchus obscurus*	[116]
AJ226120	*Globicephala macrorhynchus*	(Grohmann *et al*., 1998)
U20921	*Globicephala macrorhynchus*	[117]
U20926-U20938	*Globicephala melas*	[117]
AF056233-AF056243	*Tursiops aduncus*	[118]
AF287952-AF287955	*Tursiops aduncus*	[25]
AF355577-AF355581	*Tursiops aduncus*	(Ji *et al*., 2001)
AF459507	*Tursiops aduncus*	(Ji *et al*., 2001)
AF459518	*Tursiops aduncus*	(Ji *et al*., 2001)
AF459520-AF459521	*Tursiops aduncus*	(Ji *et al*., 2001)
AY371171-AY371177	*Tursiops truncatus (S. Australia)*	(Charlton & McKechnie, 2003)
AF056232	*Tursiops truncatus (Taiwan)*	[118]

Unpublished sequences from GenBank:

Charlton K, McKechnie SW: **Diversity of mitochondrial DNA control region of bottlenose dolphins (*Tursiops *sp.) from Southern Australian waters**. Biological Sciences, Monash University; 2003.

Grohmann L, Bokermann I, Unseld M, Hiesel R, Malek O, Giese A, Brennicke A: **Whale meat from protected species is still being sold on Japanese markets**. Berlin, Germany; 1998.

Ji GQ, Yang G, Liu S, Zhou KY: **A study on the variability of the mitochondrial DNA control region of bottlenose dolphins (genus: *Tursiops*) in Chinese waters**. Institute of Genetic Resources, College of Life Sciences, Nanjing University; 2001.

Yamagiwa D: ***Grampus griseus *(Risso's dolphin) mitochondrial d-loop region DNA**. Graduate School, Agricultural Life Sciences, The University of Tokyo, Veterinary Anatomy; 1998.

Tissue (skin or muscle) was obtained from free ranging dolphins following the methods of Gorgone *et al*. [49] or from sampling of dead, stranded individuals; a minimum of five individuals per species was included in an attempt to encompass some geographic variation that may be present in species with large oceanic distributions (Table 2). Samples from *Tursiops truncatus* included collections from both of the distinct morphotypes found in the Northwest Atlantic Ocean, described as the coastal form and the offshore form [50]. DNA from the four *T. aduncus *samples not obtained from GenBank and ten *Sousa chinensis *samples was provided by the National Marine Fisheries Service, Southwest Fisheries Science Center Marine Mammal and Sea Turtle DNA Archive (loan #113, loan #157).

**Table 2:** Number of individuals sampled across nine geographic regions for mtDNA (N_M_) and AFLP (N_A_) datasets.

	**ETP**		**NEP**		**GOM**		**WSA**		**WNA**		**ENA**	**BS**		**SA**	**IPO**		**N_mtDNA_**	**N_h_**	**N_AFLP_**
									
	**N_M_**	**N_A_**	**N_M_**	**N_A_**	**N_M_**	**N_A_**	**N_M_**		**N_M_**	**N_A_**	**N_M_**	**N_M_**	**N_A_**	**N_M_**	**N_M_**	**N_A_**			
*Delphinus capensis*			12	9													12	10	9
*D. delphis*	6	4	8	11					241	6	60	4	3				319	94	24
*Lagenodelphis hosei*					1	1			4	4							5	2	5
*Sousa chinensis*															10	5	10	5	5
*Stenella attenuata*	6	6			31	5			3								40	13	11
*S. clymene*					10	5			12	6							22	14	11
*S. coeruleoalba*	1	1			5	5			18	9							24	17	15
*S. frontalis*					76	3			123	9							199	37	12
*S. longirostris*	1	1			23	5											24	8	6
*Tursiops aduncus*														*4*	4, *19*	4	27	25	4
*T. truncatus *NW Atlantic coastal form					159	7			647	8					*1*		807	48	15
*T. truncatus *NW Atlantic offshore form					15				327	5							342	51	5
*T. truncatus *(undetermined form)														*7*			7	7	0
*Globicephala macrorhynchus *(OG)									*1*						*1*		2	2	0
*Globicephala melas *(OG)									*3*								3	3	0
*Grampus griseus *(OG)															*1*		1	1	0
*Lagenorhynchus acutus *(OG)									*1*	1							1	1	1
*Lagenorhynchus obliquidens *(OG)			*2*														2	2	0
*Lagenorhynchus obscurus *(OG)								*5*									5	5	0
*Steno bredanensis *(OG)									1	1							1	1	1
TOTAL																			
																	1853	346	124

Total number of samples (N_mtDNA_) and haplotypes (N_h_) in the mtDNA dataset, and total number of samples in AFLP dataset (N_AFLP_). Numbers in italics represent sequences from GenBank (see Table 1). Region abbreviations: ETP = eastern tropical Pacific; NEP = northeast Pacific; WSA = western South Atlantic; GOM = Gulf of Mexico; WNA = western North Atlantic; ENA = eastern North Atlantic; BS = Black Sea; SA = South Australia; IPO = Indo-Pacific Ocean; (OG) = outgroup species

### Sampling - AFLP

A total of 124 samples representing eleven delphinine species plus two outgroup species- *Steno bredanensis *(G. Cuvier in Lesson, 1828) and *Lagenorhynchus acutus *(Gray, 1828) - were included in the AFLP analysis. A minimum of four individuals per species was used and samples from different geographic areas were used when available (Table 2). Tissue (skin or muscle) was obtained as above via remote biopsy or sampling of stranded individuals. DNA from the four *T. aduncus *samples and ten *Sousa chinensis *samples was provided as above. One hundred fourteen samples from the AFLP dataset were also included in the mtDNA analysis; overlapping samples were incorporated for all ingroup taxa.

### DNA Extraction

DNA from 325 *Delphinus *spp. samples, 199 *Stenella frontalis *samples, and 111 *Tursiops truncatus *samples was extracted for previous studies [29,42,44-48]. DNA from the remaining samples (n = 1183) was extracted via the proteinase K method as described in [51] with the exception of the buffer volume (250 μL). DNA concentrations were assessed on a Hoefer DyNA Quant 200 fluorometer prior to PCR amplification and DNA quality was assessed via agarose gel electrophoresis.

### mtDNA control region sequencing

A DNA fragment approximately 450 bp in size containing the flanking proline tRNA gene and 5' end of the control region was amplified using the primers L15824 and H16265 [52]. Amplification reactions contained 50 ng of DNA, 0.3 uM of each primer, 150 uM dNTPs, 1.5 mM MgCl2, 1× PCR buffer (20 mM Tris-HCl, pH 8.4, 50 mM KCl) and 1.25 U of Taq DNA polymerase (Gibco/Invitrogen Carlsbad, CA). The thermal cycling profile started with a 30 second 94°C denaturing step followed by 35 cycles of 94°C for 30 s, 55°C for 30 s, and 72°C for 30 s. A final 10-minute extension step was added after the last cycle to extend incomplete fragments. The product was gel purified via excision from a 0.8% low melting point agarose gel followed by agarase digestion. Cycle sequencing was performed using ABI Big Dye Terminator^® ^fluorescent dye chemistry versions 1.0, 1.1, or 2.0 (Applied Biosystems, Foster City, CA) and PCR products were sequenced in both directions. Cycle sequencing products were cleaned via ethanol precipitation according to ABI protocols or with CentriSep spin column strips (Princeton Separations, Princeton, NJ). Products were sequenced via capillary electrophoresis on ABI 310 PRISM^® ^and ABI 3130 genetic analyzers. Sequence electropherograms for forward and reverse reads of each fragment were edited using Sequence Navigator 1.0.1 (Applied Biosystems, Foster City, CA) and a consensus of the two directions was constructed. The consensus sequences were truncated to control region only (362 bp) and haplotypes aligned by eye in SeqPup 0.6f [53].

### AFLP assay

The AFLP assay was run according to the protocol of Vos *et al*. [32,33] and Applied Biosystems [54] with modifications made in Kingston and Rosel [42]. *Taq*I was used as the frequent cutter enzyme rather than *Mse*I for increased resolution in C/G rich vertebrate genomes [33]. Twenty selective primer combinations were used to generate the AFLP fragments [42]. *Eco*RI selective primers were fluorescently labeled for detection on an ABI 310 PRISM^® ^genetic analyzer so only fragments containing an *Eco*RI site were detected.

### AFLP Scoring

Resulting electropherograms were scored for polymorphic peaks using Genotyper^® ^2.1 software (Applied Biosystems, Foster City, CA). Peaks were scored as dominant markers, present or absent (1 = present, 0 = absent). One base-pair (bp) sized bins were created for each dominant marker category. Markers ranged in size from 75 bp to 300 bp. We used the conservative scoring protocol described in Kingston and Rosel [42] to protect against potential problems associated with uneven amplification among samples and poor amplification of larger fragments for degraded DNA samples. Only fragments that showed even amplification across all samples were scored (no fragments where any sample exhibited poor amplification, less than 100 fluorescence units, were used) and scoring was halted at the size (usually 200-300 bp) where poorer quality samples began to lose monomorphic peaks [42].

### Analyses - mtDNA control region

Sequences were aligned by eye (362 bp) and unique haplotypes identified. A model and parameters for the phylogenetic reconstruction were determined empirically using likelihood via Modeltest 3.7 [55]. The Akaike Information Criterion (AIC) indicated that the Tamura-Nei model of DNA evolution with a gamma correction (α = 0.3409), proportion of invariable sites = 0.3735, and empirical base frequencies (A = 0.3423, C = 0.2218, G = 0.1035, T = 0.3324) was most appropriate given the data.

The alignment of control region haplotypes was analyzed in a likelihood framework using GARLI 0.96 [56]. The model and parameters determined above in Modeltest 3.7 [55] were applied in GARLI (TrN +I +G). Two replicates were performed in order to assess convergence on a topology. Stop generation and stop time were set at 5,000,000; genthreshfortopoterm was set at 20000; scorethreshforterm was set at 0.05. The remaining options were set as default. The analysis was bootstrapped for 500 iterations.

Additionally, the aligned control region haplotypes were analyzed in PAUP* 4.0b10 [57] using distance methods. Using the above model and the neighbor-joining algorithm, a phylogenetic reconstruction was rendered and bootstrapped (with replacement) using 1000 iterations. Within species, between species and corrected between species genetic distances were estimated using MEGA 3.1 and the Tamura-Nei model with parameters as described above.

The trees were rooted with a single outgroup haplotype (*Lagenorhynchus acutus*) although six more outgroup taxa (14 haplotypes) were included in the analysis (Tables 1, 2).

### Analyses - AFLP

An initial binary data matrix was compiled for all individuals and dominant AFLP markers. This presence-absence matrix was used as the basis for all AFLP analyses. Species-specific markers were defined directly from the raw binary data; a species-specific marker was shared by all individuals sampled from a particular species to the exclusion of all other taxa (synapomorphy).

Relationships among taxa and individuals were defined using a neighbor-joining phylogram [58,59] which was built using Nei-Li distance derived from the binary data matrix and bootstrapped (with replacement) 1000 times using PAUP* 4.0b10 [57]. Distance-based methods were included because the parsimony criterion, in particular, may be inappropriate for use with dominant, anonymous markers due to the inherent faulty assumption of homology among shared absent markers and the possible parsimonious, but incorrect, reconstruction in which no markers are assigned to an ancestor at a given internal node [60,61].

We also utilized the Bayesian phylogenetic inference method through MrBayes 3.1 [62,63]. Due to the binary nature of the AFLP data and the difference in probabilities between 1 to 0 and 0 to 1 state changes, we chose the restriction "noinvariantsites" option and the "noabsentsites" option. The analysis was run over 2 replicates to assure convergence on a topology; each run was performed over 10,000,000 generations (sampling at every 100 generations) and burn-in was set at 250,000 generations. The remaining options were set as default.

Non-metric multidimensional scaling analysis (NMDS) was used to further clarify and visualize relationships among taxa outside the context of a bifurcating tree. NMDS is an ordination technique designed to portray relationships as defined by a Jaccard similarity matrix in three-dimensional space. A Jaccard similarity matrix was created from the initial binary data using NTSYS-pc [64]. Jaccard similarity values range from 0 (no similarity) to 1 (identical) and are based on shared presence of markers:



where *a *is the number of polymorphic markers shared by individuals *x *and *y*, *b *is the number of markers present in *x *but absent in *y*, and *c *is the number of markers present in *y *but absent in *x *[65].

NMDS plots were created using NTSYS-pc [64]; three sets of principal coordinates analysis values were used as the initial configuration for better fit. The goodness of fit of the NMDS model was measured using a stress value ranging from 0-1 (0 = excellent fit, 1 = poor fit).

### Interspecies hybrids

During the study, four spotted dolphin individuals (*Stenella frontalis *and *S. attenuata*) were genetically identified as possible interspecies hybrids due to incongruence between field identification, mtDNA haplotype and placement within the phylogenetic reconstruction based on the AFLP markers. These four individuals were added to the core AFLP data set in separate, identical phylogenetic analyses repeated as above (both Bayesian and distance methods rendered nearly identical results with regard to the putative hybrids).

We then utilized the program STRUCTURE 2.2 on the multilocus AFLP data to assess hybrid origin and perform assignment tests for the putative hybrids [66,67]. We first ran STRUCTURE using the AFLP genotypes of what we considered pure *S. attenuata *(n = 12) and *S. frontalis *(n = 11) samples to confirm the number of 'populations' and their composition without the putative hybrids. These data were analyzed via the admixture model, independent alleles, 10,000 burn-in and 100,000 MCMC, three replicate runs at each K (1-5), lambda = 1, recessive alleles option, and no prior population information. This test was repeated with inferred lambda as well as under the no admixture model. Next, the hybrids were added and prior population info was used for the 23 "known" *S. attenuata *and *S. frontalis *samples; this dataset was used to identify affinities of each of the four putative hybrids to either species. This analysis was run under both admixture and no admixture models, 10,000 burn-in and 100,000 MCMC, migration 0.05. Finally, that same analysis was run without prior population info for all samples included in order to examine probabilities of alternative parentage and grandparentage.

## Results

### mtDNA control region

The 1808 delphinid sequences - 606 sequenced for previous studies (GenBank accession numbers AY997307 - AY997311, DQ060054 - DQ060064, U01956, U02639 - U02664, FM211489-FM211508, FM211510-FM211511, FM211513-FM211563, GQ504040-GQ504057) and 1202 sequenced in this study - resulted in 303 unique 362 bp mtDNA control region haplotypes. With the addition of outside sequences from GenBank, a total of 346 haplotypes were present in the alignment (Table 2). Two *T. aduncus *sequences (Indo-Pacific-Taiwan) from our study matched the downloaded GenBank haplotypes AF459507 and AF459518. Haplotypes from our study submitted to GenBank bear accession numbers DQ845437-DQ845453 and GQ504058-GQ504195. The maximum within-species diversity was equal to or exceeded net between-species distances in 24 of 55 (44%) pairwise comparisons (Table 3). Net mean between-species distance ranged from 0.013-0.093 while mean within-species distances ranged from 0.005 to 0.030.

**Table 3:** Genetic distance within and between Delphininae species based on mtDNA control region sequences and the Tamura-Nei model of evolution.

	**Tt**	**Ta**	**Dc**	**Dd**	**Sa**	**Sf**	**Sco**	**Scl**	**Sl**	**Lh**	**Sch**
*Tursiops truncatus*	*0.030 [0.007]*	0.066 [0.016]	0.053 [0.013]	0.049 [0.011]	0.077 [0.018]	0.054 [0.013]	0.042 [0.010]	0.058 [0.014]	0.053 [0.012]	0.061 [0.016]	0.078 [0.018]
*Tursiops aduncus*	0.041 [0.012]	*0.020 [0.005*]	0.064 [0.017]	0.056 [0.014]	0.112 [0.029]	0.055 [0.015]	0.054 [0.014]	0.063 [0.017]	0.068 [0.017]	0.085 [0.023]	0.095 [0.023]
*Delphinus capensis*	0.031 [0.011]	0.047 [0.015]	*0.014 [0.004]*	0.033 [0.009]	0.075 [0.019]	0.040 [0.012]	0.040 [0.011]	0.053 [0.015]	0.051 [0.013]	0.067 [0.019]	0.081 [0.020]
*Delphinus delphis*	0.024 [0.008]	0.036 [0.011]	0.016 [0.008]	*0.021 [0.005]*	0.065 [0.016]	0.031 [0.008]	0.032 [0.008]	0.043 [0.011]	0.042 [0.010]	0.052 [0.014]	0.070 [0.017]
*Stenella attenuata*	0.051 [0.015]	0.091 [0.025]	0.057 [0.017]	0.044 [0.013]	*0.022 [0.005]*	0.070 [0.018]	0.068 [0.017]	0.090 [0.024]	0.054 [0.013]	0.061 [0.016]	0.106 [0.026]
*Stenella frontalis*	0.030 [0.010]	0.037 [0.012]	0.026 [0.010]	0.013 [0.005]	0.051 [0.015]	*0.016 [0.004]*	0.037 [0.010]	0.044 [0.012]	0.047 [0.012]	0.060 [0.017]	0.077 [0.020]
*Stenella coeruleoalba*	0.014 [0.006]	0.032 [0.011]	0.021 [0.008]	0.009 [0.004]	0.044 [0.013]	0.016 [0.007]	*0.026 [0.006]*	0.038 [0.010]	0.043 [0.011]	0.045 [0.012]	0.082 [0.021]
*Stenella clymene*	0.030 [0.011]	0.041 [0.013]	0.035 [0.012]	0.020 [0.007]	0.066 [0.019]	0.024 [0.009]	0.013 [0.006]	*0.024 [0.006]*	0.056 [0.015]	0.061 [0.017]	0.086 [0.022]
*Stenella longirostris*	0.024 [0.010]	0.045 [0.014]	0.031 [0.011]	0.018 [0.007]	0.029 [0.010]	0.025 [0.009]	0.016 [0.007]	0.030 [0.011]	*0.027 [0.007*]	0.050 [0.013]	0.086 [0.021]
*Lagenodelphis hosei*	0.035 [0.013]	0.064 [0.019]	0.050 [0.017]	0.031 [0.012]	0.039 [0.014]	0.041 [0.015]	0.022 [0.009]	0.038 [0.013]	0.026 [0.011]	*0.022 [0.010]*	0.079 [0.021]
*Sousa chinensis*	0.061 [0.018]	0.083 [0.023]	0.072 [0.020]	0.057 [0.016]	0.093 [0.025]	0.066 [0.019]	0.067 [0.019]	0.072 [0.020]	0.070 [0.020]	0.066 [0.020]	*0.005 [0.003]*

Net mean between species distance below diagonal, mean within species distance along diagonal (italics), uncorrected mean between species distance above diagonal. In brackets, standard error based on 500 bootstrap replicates.

**Figure 2:**
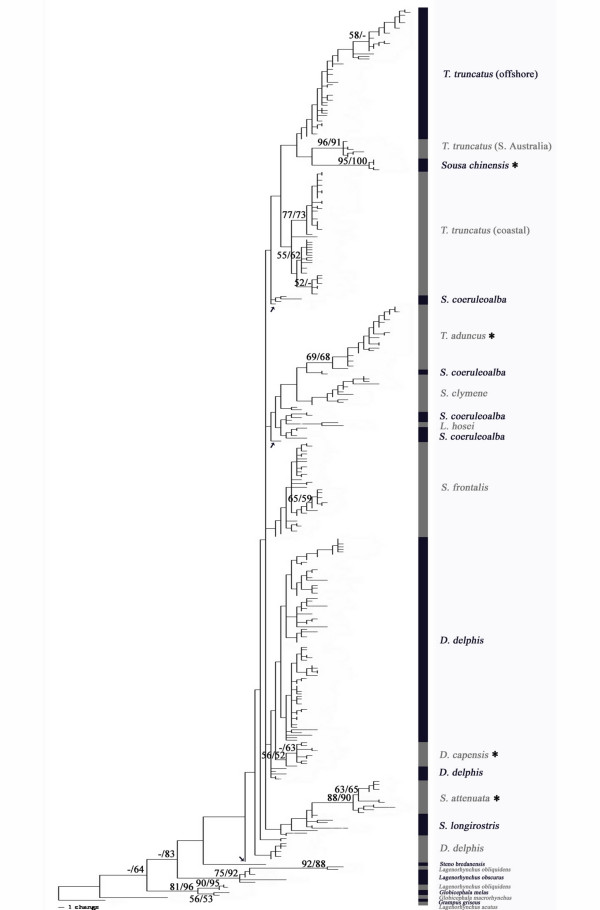
**Phylogenetic analysis of mtDNA control region haplotypes**. Phylogenetic tree (best tree) inferred from mtDNA control region haplotypes using the maximum likelihood tool GARLI. Tree is rooted with the outgroup *Lagenorhynchus acutus*. ML bootstrap values (500 replicates) greater than 50% are denoted as the first number above supported nodes, NJ/Tamura-Nei bootstrap values (1000 replicates) greater than 50% are denoted as the second number. Supported nodes joining just a few terminal taxa only are not listed for clarity. Haplotypes of species denoted by an asterisk (*) group to monophyletic clades with bootstrap support greater than 50%. Arrows denote haplotypes that fall outside any coherent species clade.

The phylogenetic trees derived from the mtDNA control region data reveal that although some species form monophyletic groups (*Tursiops truncatus *coastal, *T. truncatus *offshore, *T. aduncus*, *Stenella attenuata, Delphinus capensis, Sousa chinensis*), these nodes are not unilaterally supported after bootstrap resampling (Figure 2). *Stenella attenuata *and *Sousa chinensis *are the only species that show robust bootstrap support (Figure 2). The control region trees offer little resolution with regard to branching order among species even without bootstrap support. Several *D. delphis *and *S. coeruleoalba *haplotypes fall outside any coherent species clade (Figure 2, haplotypes marked with arrows).

### AFLP

The AFLP assay rendered 418 total polymorphic markers among 124 individuals representing 14 species. Each primer combination exhibited on average 20.90 ± 7.22 polymorphic markers (mean ± SD). Each individual exhibited on average 61.26 ± 6.10 (mean ± SD) polymorphic markers when all primer combinations were considered. Five of the 418 polymorphic characters were species-specific markers as they demonstrated species-level synapomorphies.

**Figure 3:**
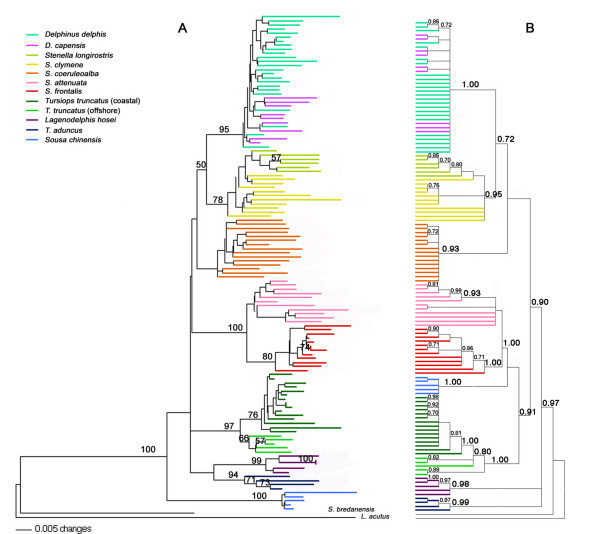
**Phylogenetic trees inferred from AFLP data for the subfamily Delphininae**. Species are indicated by color-coded branches. Trees are rooted with the outgroup *Lagenorhynchus acutus*. A. Nei-Lei neighbor joining analysis; bootstrap values over 50% from 1000 iterations are noted on nodes. B. Majority rule consensus tree from Bayesian phylogenetic reconstruction (MrBayes); posterior probabilities above 0.70 are noted on nodes.

Both AFLP phylogenies are more resolved than the phylogenies derived from the mtDNA data, although branching order among some of the deeper nodes is not determined (Figure 3A &3B). The Bayesian analysis of the AFLP data resolves the deeper nodes best. Both *D. capensis *and *S. longirostris *are nested within taxa, *D. delphis *and *S. clymene*, respectively. The two spotted dolphin species, *S. frontalis *and *S. attenuata*, are joined with very high support, although on a species level, only *S. frontalis *is monophyletic upon bootstrapping. These sister species do not cluster exclusively with other *Stenella *species, rendering the genus polyphyletic (or paraphyletic due to the placement of the genus *Delphinus *in the neighbor-joining tree). *Tursiops truncatus *coastal and offshore morphotypes from the Atlantic Ocean form two distinct groups; the node joining these two sister taxa exhibits excellent support. An unsupported node clustering the rest of the subfamily suggests *Sousa chinensis *is the sister taxon to the rest of the delphinines in the distance analysis. However, the placement of *Sousa chinensis *is the most notable point where the two analyses disagree; the Bayesian tree suggests this species clusters with the spotted dolphins and *T. truncatus*. The entire subfamily clusters to the exclusion of *Steno bredanensis *with excellent bootstrap support in both reconstruction methods.

**Figure 4:**
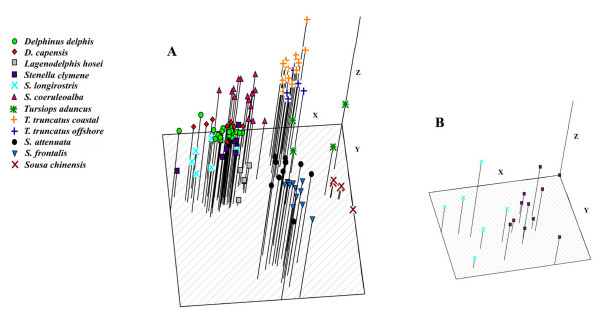
**NMDS analysis of AFLP data**. A. Non-metric multidimensional scaling (NMDS) analysis plot of AFLP data from 122 delphinine individuals from 11 species. Stress = 0.32 B. NMDS analysis plot of only *S. clymene *and *S. longirostris *individuals. Stress = 0.19.

Non-metric multidimensional scaling analysis reveals large-scale differences between the sister taxa *S. frontalis *and *S. attenuata *and the rest of the Delphininae (Figure 4A). The genus *Tursiops *is slightly less distant from the remaining delphinines. *Stenella coeruleoalba*, *L. hosei*, and *Sousa chinensis *form distinct species groups, while the overlapping *S. longirostris *and *S. clymene *clusters associate closely with *Delphinus *spp. Although *S. longirostris *and *S. clymene *cannot be distinguished on the delphinine NMDS plot due to the variance among *S. clymene*, when the NMDS analysis was run for only these taxa, the two species formed distinct groups (Figure 4B). The same has been demonstrated for sister taxa pairs *D. delphis*, *D. capensis*, as well as coastal and offshore morphotypes of *T. truncatus *[42]. *Tursiops aduncus*, although represented by only four individuals, does not form a tight species cluster. Two of the four *T. aduncus *representatives associate more closely with the *T. truncatus *group than any other group.

### Interspecies hybrids

Initial STRUCTURE analyses confirmed two 'populations' (K = 2: (average lnP(D) for K = 1-5: -1361.7, -1124.2, -1205.5, -1329.3, -1481.0, respectively) from the AFLP data (excluding putative hybrid data) corresponding to the two discrete species (*S. attenuata *and *S. frontalis*). Probabilities of identity of the four putative hybrids based on the AFLP data were opposite the species identifications inferred from the mtDNA data, but congruent with field identifications based on external morphological characters (Table 4). An additional hybrid, for which the mtDNA, field data and AFLP data are all congruent, may also have been identified.

**Table 4:** Summary of hybrid morphological and genetic characteristics and results of STRUCTURE assignment and ancestry analyses.

				**Assignment probability with prior information**		**Assignment probability without prior information**	
				
**Sample**	**Field ID**	**mtDNA haplotype**	**AFLP clade**	**prob *S. attenuata***	**prob *S. frontalis***	prob parent*	prob grandparent*
2Sf01	*S. attenuata*	*S. frontalis*	*S. attenuata*	0.972	0.028	0.000	0.000
D2BC122	*Stenella sp.*	*S. attenuata*	*S. frontalis*	0.040	0.960	0.000	0.000
D2BC75	*S. frontalis*	*S. attenuata*	*S. frontalis*	0.047	0.953	0.000	0.000
Sa99270	*S. frontalis*	*S. attenuata*	*S. frontalis*	0.022	0.978	0.000	0.000
Sa94106	*S. attenuata*	*S. attenuata*	*S. attenuata*	0.734	0.266	0.008	0.730

* Posterior probability that each individual has recent ancestry (parental or grandparental) from the species opposite that identified by the AFLP analysis.

Two STRUCTURE assignment and ancestry analyses were performed, one with prior population information assigned and the second with putative hybrid population identity set as unknown to examine alternate parentage contributions. Last row identifies fifth possible hybrid discovered by the analysis.

## Discussion

### Mitochondrial versus AFLP phylogenies

Traditional methods of phylogenetic inference using a single gene, or even several genes, often yield a limited picture of evolutionary history for closely related species. By focusing on a scale so fine and specific, discrepancies between loci are the norm [68-70]. The difficulty of finding a gene that reflects complete lineage sorting, yet is variable enough to reliably untangle relationships between closely related taxa that are the product of a recent radiation event is apparent in many phylogenetic studies [71]. With the advent of more powerful analyses that consider hundreds of nuclear markers, the resolution and power of phylogenetic analyses has drastically increased [72,73]. Here we compared the ability of two different molecular marker types, mtDNA sequences and AFLP markers, to reconstruct the evolutionary history among a group of recent and likely rapidly radiated taxa.

The comparison of mitochondrial and AFLP phylogenies reveals congruence in some cases but discrepancies in other inferences concerning the relationships among members of the subfamily Delphininae. Overall, the mtDNA control region phylogeny, similar to previously published cytochrome b phylogenies [3,24,25], offers little power for resolving relationships among these delphinine taxa (Figures 1, 2). In the control region, the high levels of within species diversity, often equal to or exceeding levels of between-species divergence (Table 3), may significantly interfere with the ability to construct a robust phylogeny. In some cases, pairwise between-species distances are less than the distance between two individuals of the same species. The AFLP reconstructions provide stronger support for many relationships within the subfamily and untangle other relationships that mtDNA leaves unresolved (Figure 3).

Discrepancies between mitochondrial and nuclear phylogenies are not unique to this study [74,75]. Although both sets of data exhibit a pattern of high levels of intraspecific variation and low levels of interspecies divergence, phylogenetic inference from the mtDNA data in particular is hindered by this pattern. One explanation for this pattern is a recent, rapid radiation, with incomplete lineage sorting of mtDNA control region haplotypes among the delphinine species [76]. The occurrence of the marked haplotypes that do not cluster together with conspecifics (Figure 2) could be explained by such a hypothesis. The fact that all of the "misplaced" haplotypes in the mtDNA phylogeny group with the expected species in the AFLP tree indicates they were not simply misidentified samples. Although this pattern might also arise if there were a recent nuclear duplication of this mitochondrial gene [77,78], there is no evidence to date of such a control region duplication in cetaceans. Whatever the cause, the mtDNA control region tree alone is insufficient for robust phylogenetic inference in this subfamily. Although the resolution for individual species groups is better in the AFLP trees, the deeper internodes are short and not consistently well-supported. This short internode congruence with the mtDNA pattern suggests rapid radiation over other alternatives.

In the mtDNA analysis, few species form monophyletic groups in the control region phylogeny and only two species, *Sousa chinensis* and *Stenella attenuata*, render monophyletic species clades consistently supported. While it is possible to use the mtDNA control region sequence for species identification if an unknown falls into one of these groups, or even a monophyletic species clade lacking bootstrap support (e.g. *T. truncatus*), interpretation is much less clear if an unknown haplotype falls outside a coherent monophyletic group. Use of the AFLP markers appears to be a more consistent choice for species identification when using phylogenetic methods. It has proven successful in identification of maple species and individuals [79]. Even in the dolphin species, which have considerably lower levels of nuclear variability [80,81], we found no two delphinine individuals to have identical AFLP profiles; this specificity suggests the method may prove useful for identifying individuals. However, the methodology is considerably more labor intensive than DNA sequencing of an mtDNA fragment, and may prove less useful in cases of highly degraded tissue samples.

Given the low divergence among taxa (exhibited by short internodes) and high levels of diversity within species, it is not entirely surprising the single locus mtDNA phylogenetic reconstructions offer little resolution. Due to the prevalence of mitochondrial control region data collected, as well as the assertion that mitochondrial markers alone are excellent in phylogeographic context [82], we felt it necessary to demonstrate this evolutionarily interesting example in which the data fail. Because there is so much morphological variation within delphinine species, one goal of this study was thorough taxon sampling. Since the great majority of the variation in the control region is limited to the region we amplified [83], one might be concerned about resolving power for so many individuals over the short stretch of DNA. If the number of taxa is pruned along the branches of the tree, we still observe the same poor resolution (see Additional file 1).

### Interspecies hybrids

Interspecies hybrids among delphinid taxa have been recorded, but most documented cases come from animals in captivity rather than in the wild [84-88]. In our study, the combined mtDNA and AFLP data, along with corresponding morphological field identifications, indicate at least four inter-species hybrids between the two spotted dolphin species.

These two species, although morphologically similar [26], exhibit ten fixed differences among mtDNA control region haplotypes [45]. The *S. attenuata *group is one of only two species exhibiting strong bootstrap support in the mtDNA trees. Although the resolution is generally poor in the mtDNA phylogenies, we can at least reliably discern *S. attenuata *from all other delphinines based on mitochondrial data alone. In addition, none of the putative hybrid haplotypes are unique, i.e., they are shared by other individuals of that species in the data set.

**Figure 5:**
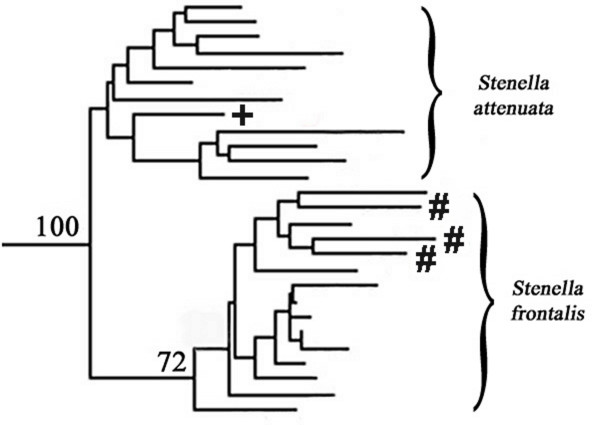
**Inset of *Stenella attenuata *+ *S. frontalis *clade if putative hybrid samples are added to the AFLP Nei-Li (NJ) phylogenetic analysis**. The topology of the remainder of the tree (not shown) is identical to Figure 3A. Individuals denoted with # exhibit *S. attenuata *mtDNA control region haplotypes; individual denoted with + exhibits a *S. frontalis *mtDNA control region haplotype. These putative hybrid individuals fall in clades in the AFLP tree opposite their mtDNA identity.

In an AFLP phylogenetic reconstruction, the hybrid sample exhibiting a *S. frontalis *mtDNA control region haplotype sits in a monophyletic clade with the *S. attenuata*, while the three hybrid samples bearing *S. attenuata *mtDNA control region haplotypes fall inside the bootstrap-supported *S. frontalis *clade (Figure 5). The mostly nuclear AFLP data are concordant with field identification of the samples but conflict with mtDNA haplotypes. This pattern suggests we have detected hybridization in the wild in both directions, with possible backcrossing into the paternal species. It is important to note we would likely not be able to detect backcrossing of hybrids into the maternal species, at least with mtDNA sequence incongruence.

The STRUCTURE assignment and ancestry tests on the AFLP data confirm the AFLP phylogenetic results (Table 4). The results of the probability of assignment of each putative hybrid to species strongly conflicts with the mtDNA haplotype identity, suggesting hybrids are backcrossed many generations into the paternal species. We also see an interesting result with the *S. attenuata *individual Sa94106. This individual shows a low probability of assignment to the *S. attenuata *species but rather than a high probability of assignment in *S. frontalis*, this individual exhibits high probability of mixed grandparentage (Table 4). This individual may represent the category of hybrids mentioned in the previous paragraph, those backcrossed into the maternal species. Even some of the unambiguous individuals show non-zero probabilities for extra-species grandparentage, suggesting low levels of allelic introgression may be widespread.

Hybridization and its evolutionary role have been recently revisited in the literature now that larger nuclear datasets are increasingly available for comparison to mtDNA phylogenies [75,89-94]. Shaw [75] demonstrated the phenomenon of mtDNA gene flow and in some cases complete introgression (haplotype capture) across species boundaries in the Hawaiian cricket genus *Laupala*. The apparent importance of interspecific gene flow in this system led Shaw [75] to issue a caveat about potentially misleading patterns of mtDNA variation among closely related species complexes. Among species of Darwin's finches in the Galápagos archipelago, sympatric introgressive hybridization has also played an important role in the adaptive radiation of species [91]. It is now recognized that interspecific hybridization in the wild is not uncommon in rapidly radiating groups [95]. A reader might form an intuitive hypothesis regarding the detection of hybridization in wild populations: hybridization levels must be relatively high if even a handful of hybrids are detected by chance. Therefore, species identities should be lost over time due to gene flow. While this may be a possible fate for some lineages through the course of evolutionary history, there is a myriad of recent literature documenting cases of just the opposite: divergence with gene flow and long-term maintenance of species boundaries in the face of secondary contact [89,90,92-94,96]. The highly labile spotted dolphin species may demonstrate a stable hybrid zone across yet to be quantified gradients or patches in marine variables such as salinity, temperature, depth, or prey distribution. Differential introgression of loci across the genome between species due to hybridization is possible and common; gene flow and species divergence are not always mutually exclusive trajectories [89,90,94].

Although we cannot make definitive conclusions about the role of hybridization in the evolution of the delphinine species, it is important to note the evolutionary similarities among the Delphininae, the *Laupala*, and the Darwin's finches. The Delphininae, like these other taxa, are likely the product of a recent, rapid radiation event. In addition, many of the species in the subfamily are distributed across ocean basins. In contrast with island species, it is more difficult to discern obvious barriers to gene flow in marine species. However, isolated allopatric or parapatric populations exist within delphinine species that are in turn sympatric with other delphinine species [44,97]. These features outline a system in which rare, interspecies, introgressive hybridization could possibly play an evolutionary significant role among the delphinines. At the very least, the occurrence of interspecies hybridization in the wild draws into question the practice of species identification for some delphinines based on mtDNA sequences alone.

### Systematic relationships inferred from AFLP data

The taxonomy and systematics of the family Delphinidae have been unresolved for centuries. Rice [98] recognized 36 species, but new species of *Sotalia *and *Orcaella *have recently been described [20,28] and additional species will likely be described in coming years [25,99,100]. It is clear that the biodiversity of this family remains underestimated at this time. At the subfamily level, as few as two and as many as five subfamilies have been proposed [98], but the author suggests that subfamily designations are "best held in abeyance" pending further studies [98]. The subfamily Delphininae, however, has maintained itself across the variant classifications with major conflicts of membership confined to the genera *Grampus*, *Steno*, *Sousa *and *Sotalia *[3,20,101]. It seems generally agreed that *Grampus *belongs in the subfamily Globicephalinae [3,98,101], that *Sousa *belongs within the Delphininae [3,20,24], and *Sotalia *may be at least a sister lineage [20].

However, to date, no comprehensive morphological or molecular study has had sufficient power to satisfactorily resolve the relationships among the species in the Delphininae. Most analyses do not have dense within taxon sampling or complete taxon sampling for the entire group [3,20,21,24]. Some recent single-marker analyses aimed at larger phylogenetic groups may appear to provide some resolution of internal nodes within the subfamily Delphininae [21,24]. However, when a single haplotype represents each species, we are simply looking at a gene tree among the single lineages chosen by chance from those species' wide geographic distributions. As Figure 1 demonstrates, the stochastic sampling of a single haplotype per species could render a myriad of different results, some more resolved than others, but none alone particularly accurate at estimating the true course of the species' evolutionary history. To avoid the possibility of this particular confounding possibility, the comprehensive within species sampling scheme for all the Delphininae was implemented in our study. As mentioned above, even complete taxon sampling encompassing geographic variability within species does not improve the mtDNA phylogeny based on control region sequences. However, the Bayesian analysis of the AFLP data, which provide many loci spread across the genome, provides a more resolved phylogeny for the subfamily in which many relationships are congruent with previously described relationships based on morphological evidence [26,102-104]. We believe the evidence points to a real incongruence between the gene history of the mitochondrion and the evolutionary history of these recently diverged species.

Morphological data support a sister taxon relationship between the two spotted dolphin species. *Stenella frontalis *and *S. attenuata *overlap in every morphological character analyzed by Perrin *et al*. [26] with the exceptions of total vertebral count and color pattern (although this is a subtle difference). Although there are no fixed differences in skull morphometrics, the two species can be differentiated using simultaneous discriminant analysis of multiple characters [26]. Neither the mtDNA control region nor the most comprehensive cytochrome *b *[3] phylogenies indicate these two species share a most recent common ancestor relative to other *Stenella *species. In the mtDNA control region phylogeny, *S. attenuata *haplotypes are so distinct that they are the only widely sampled species within the Delphininae supported by a bootstrap value approaching 100%, but they never cluster with *S. frontalis*. In contrast, the AFLP data recover a relationship congruent with the morphological evidence with bootstrap support of 100%, suggesting that the morphological overlap may be a result of homology rather than convergence (Figure 3). The detection of hybridization between the two species (see above) also argues for a recent shared evolutionary history.

The AFLP phylogeny is the first molecular study to suggest that *S. clymene *and *S. longirostris *are more closely related to each other than any other taxa in the genus *Stenella*. This relationship is supported by morphological data as well [27,103,104]. *Stenella clymene *was only officially recognized as a valid species in 1981 when Perrin *et al*. [27] examined a series of skulls and photos of *S. clymene*, *S. longirostris *and *S. coeruleoalba*. Prior to this, the uncertainty in the validity of the species likely resulted from the fact that external color patterns in *S. clymene *resemble *S. longirostris *while the shape of the skull more closely resembles *S. coeruleoalba *[27]. By examining a series of skulls of all three taxa, Perrin *et al*. [27] determined that *S. clymene *is a valid species and concluded it is most closely related to *S. longirostris*. Interestingly, only *S. clymene *and *S. longirostris *are known to exhibit aerial spinning behavior and Perrin [102] has suggested this may represent a synapomorphic character. The AFLP data support this close relationship, in fact, with the *S. longirostris *samples being nested within the *S. clymene *clade, suggesting a recent common ancestry for these two taxa. This result contrasts with the LeDuc *et al*. cytochrome *b *phylogeny [3] that grouped *S. clymene *with *S. coeruleoalba*. The authors suggested *S. clymene *may be of hybrid origin from parental species *S. coeruleoalba *and *S. longirostris*. However, the AFLP phylogeny does not place *S. clymene *intermediate to the other two species and in fact, groups *S. clymene *with *S. longirostris *and *Delphinus *to the exclusion of *S. coeruleoalba*, thereby arguing against such a hybrid origin.

In addition, Perrin and Mead [104] suggested a close relationship between *S. clymene*, *S. longirostris *and *Delphinus*, *S. coeruleoalba *and *Lagenodelphis hosei*. The multidimensional scaling analysis of the AFLP data mirrors the morphological inferences (Figure 4); the phylogenetic analysis supports a relationship among *S. clymene *and *S. longirostris *and *Delphinus*, with *S. coeruleoalba *as the next most closely related species to this group. However, *L. hosei *is sister to all the delphinines except *T. aduncus *in both AFLP trees, a result in conflict with the morphological data as well as the mtDNA and nuclear data in Caballero *et al*. [20]. The Caballero *et al*. analysis of combined genes suggests, like the morphology, a close relationship between *L. hosei *and *D. delphis *and *S. longirostris*. However, the support for this node is moderate (low for nuclear genes alone) and five of the eleven species in the subfamily are not included in their molecular analysis [20]. Further work is needed to understand the conflicting position of *L*. *hosei*.

Contrary to morphological evidence, most molecular studies [3,20,105] and the AFLP phylogenies all suggest that the genus *Tursiops *is polyphyletic. In the AFLP tree, the monophyletic coastal and offshore *T. truncatus *clades show substantial divergence, but share a common ancestor to the exclusion of all other delphinine taxa (Figure 3). The divergence between the coastal and offshore morphotypes of *T. truncatus *has been previously documented [42,106]. This divergence is greater than the divergence seen between two recognized species *D. delphis *and *D. capensis *in both mtDNA and AFLP data and may represent species level differentiation [42]. There is no support for a close relationship between *T. truncatus *and *T. aduncus*, the other member of the genus; surprising given the morphological similarities between the two species. Recent genetic studies have suggested the existence of additional species within the genus *Tursiops *[25,107]. Given the polyphyletic nature revealed by molecular analyses, this genus deserves further investigation.

In addition, the AFLP data do not provide strong evidence for monophyly of the genus *Stenella*. The validity of this genus as a coherent evolutionary lineage has been questioned previously on both morphological and molecular grounds [3,20,24,105,108]. While nuclear, mtDNA sequence data and AFLP data do not support monophyly for this genus, the relationships among *Stenella *species and other members of the subfamily do differ significantly between the data types. The Bayesian analysis of the AFLP data is most in line with morphological inferences. The Bayesian analysis groups *S. longirostris *and *S. clymene *with the two *Delphinus *species while the two spotted dolphins species are found together in a well supported group with *T. truncatus *and *Sousa chinensis *(Figure 3B). These relationships are congruent with morphological evidence, which supports a close relationship between members of the genus *Tursiops*, *S. attenuata*, and *S. frontalis *[26,109]. Perrin *et al*. [26] describe a suite of cranial characters that differentiate *T. truncatus*, *S. frontalis *and *S. attenuata *from other delphinines. *Stenella frontalis *and *T. truncatus *also share a similar ground coloration pattern [26].

All molecular data sets do support monophyly of the genus *Delphinus *[3,24]. In the AFLP phylogenies, the *D. capensis *samples are nested within the *D. delphis *clade. The recent divergence between the two *Delphinus *species has been demonstrated in previous studies using both mtDNA and morphological data [3,23,29,42]. The fact that *D. capensis *is nested within *D. delphis *in the genome-wide survey as well as in the mtDNA phylogenies further supports the hypothesis of incipient speciation in *D. capensis*; reciprocal monophyly has not yet been attained, while at the same time there is no evidence of hybridization between these species even where they are sympatric [23].

One major inconsistency in the AFLP analyses is the placement of *Sousa chinensis*. The distance-based approach positions this species on a fairly long branch but an unsupported node placed outside of the remaining Delphininae taxa (Figure 3A). The Bayesian analysis of the AFLP data positions *Sousa *firmly within the Delphininae cluster, grouping it with *T. truncatus *and the two spotted dolphin species with posterior probability of 91% (Figure 3B). While this placement is the main difference between the two topologies, the incongruence is not drastic. The main backbone of the distance-based tree is composed mostly short internodes and little bootstrap support. While the Bayesian inference offers greater resolution, the linking of *Sousa *as a sister lineage to the pair *Stenella frontalis *and S. *attenuata *alone bears a low posterior probability. The genus *Sousa *has historically been considered a more primitive taxon grouped with genera generally considered outside the subfamily Delphininae (*Steno *and *Sotalia*: [19,110,111]). As noted by Leduc *et al*. [3], Arnold and Heinsohn [112] suggested similarities in morphological characters among *Sousa*, *Tursiops *and *Stenella*, perceiving more derived characters in *Sousa *than previous investigators. The Bayesian analysis of the AFLP data would support this hypothesis. Neither analysis, however, suggests that this genus belongs outside the subfamily, supporting other molecular studies using mtDNA and nuclear genes [20]. Investigating further the relationship between the *Sotalia *lineage and the Delphininae with complete species coverage and dense within species sampling may help clarify the placement of the genus *Sousa *[20].

## Conclusion

### Rapid radiation and implications for phylogeny reconstruction

Although there are still ambiguities to be resolved concerning relationships among some of the taxa in the subfamily Delphininae, the comparison of extensive mtDNA and AFLP datasets and their resultant phylogenies offers considerable insight into this enigmatic group. All molecular data to date support the hypothesis of a recent, rapid radiation in the evolutionary history of these taxa. The support for a rapid radiation in this subfamily is of considerable interest. How did it come about? Changes in sea level and concomitant changes in water temperatures are often suggested as having played a significant role in speciation of cetaceans [7,27,69,113]. Such changes could explain the Atlantic endemics *S. clymene *and *S. frontalis*, as described earlier. Why these species did not expand outside the Atlantic is not clear. However, currents around the Cape of Good Hope would favor movement into the Atlantic rather than out. It is more difficult to explain the rest of the Delphininae, where many species are both sympatric and distributed circumglobally. It is clear, however, that the oceans support a diverse array of similar dolphin species and hence must have produced conditions necessary for the diversification seen in this subfamily. Careful, comprehensive analyses of habitat and diet, as has been recently done with beaked whales [114], may provide insight into the different processes and pressures that produced these closely related dolphin taxa.

The pattern of high intraspecific variation and low interspecies divergence exhibited by the delphinines can be problematic when dealing with single-locus mtDNA phylogenetic reconstruction. Bootstrapping of mtDNA datasets results in nearly completely unresolved phylogenies that are highly unstable when changes in outgroup, or even ingroup, taxa are made (data not shown). Hence, inferences about the evolutionary relationships among the delphinine species cannot be made from these reconstructions. The multi-locus approach using AFLP markers offers far greater resolving power in the face of this pattern. Given the reduced effective population size of a mitochondrial marker, we often expect better phylogenetic resolution from mtDNA data. In this case, we find the opposite. Since the mitochondrion bears the evolutionary history of a single molecule nested within a species, the signal embedded, while often powerful, must be interpreted with caution. The AFLP markers are a cross section of markers across the genome, providing the phylogenetic reconstruction with a signal that is integrated across sites likely to be neutral, selected, linked to selected sites, and everything in between; the signal incorporates heterogeneity in gene histories. It is from this attribute that the assay may draw resolving power.

Finally, the AFLP data when coupled with mtDNA sequence data provide evidence of interspecies hybridization. If interspecies hybridization plays a part in delphinine evolution, even if rare in frequency, extreme caution must be used when inferring phylogeny from mtDNA loci in the absence of corresponding multi-locus nuclear data. Considering the extensive process of developing sequence-based, single-copy nuclear markers useful for phylogenetic reconstruction on this scale [71], the multi-locus AFLP approach offers us a powerful tool with which to begin addressing the problems associated with phylogenetic inference in closely related, recently diverged taxa.

## Authors' contributions

SEK, LDA, and PER all carried out aspects of the molecular labwork, data analyses, and manuscript composition. SEK ran the bulk of the AFLP data collection and analyses, while LDA and PER ran much of the sequence data collection and analyses. LDA completed the STRUCTURE analyses. SEK executed the GARLI analyses. PER was the primary force behind the study concept. All authors read and approved the final manuscript.

## Supplementary Material

Additional file 1 **Phylogenetic analysis of truncated dataset**. Phylogenetic tree (best tree) inferred from a pruned dataset of mtDNA control region haplotypes (86 taxa) using the maximum likelihood tool GARLI. Tree is rooted with the outgroup *Lagenorhynchus acutus*. ML bootstrap values (100 replicates) above 50% are denoted above supported nodes. Click here for file
