# Supplementary figures and images for: Testing mitochondrial sequences and anonymous nuclear markers for phylogeny reconstruction in a rapidly radiating group: molecular systematics of the Delphininae (Cetacea: Odontoceti: Delphinidae)
Source: BMC Evol Biol. 2009 Oct 7;9:245. doi: 10.1186/1471-2148-9-245 (PMC2770059; doi:10.1186/1471-2148-9-245)

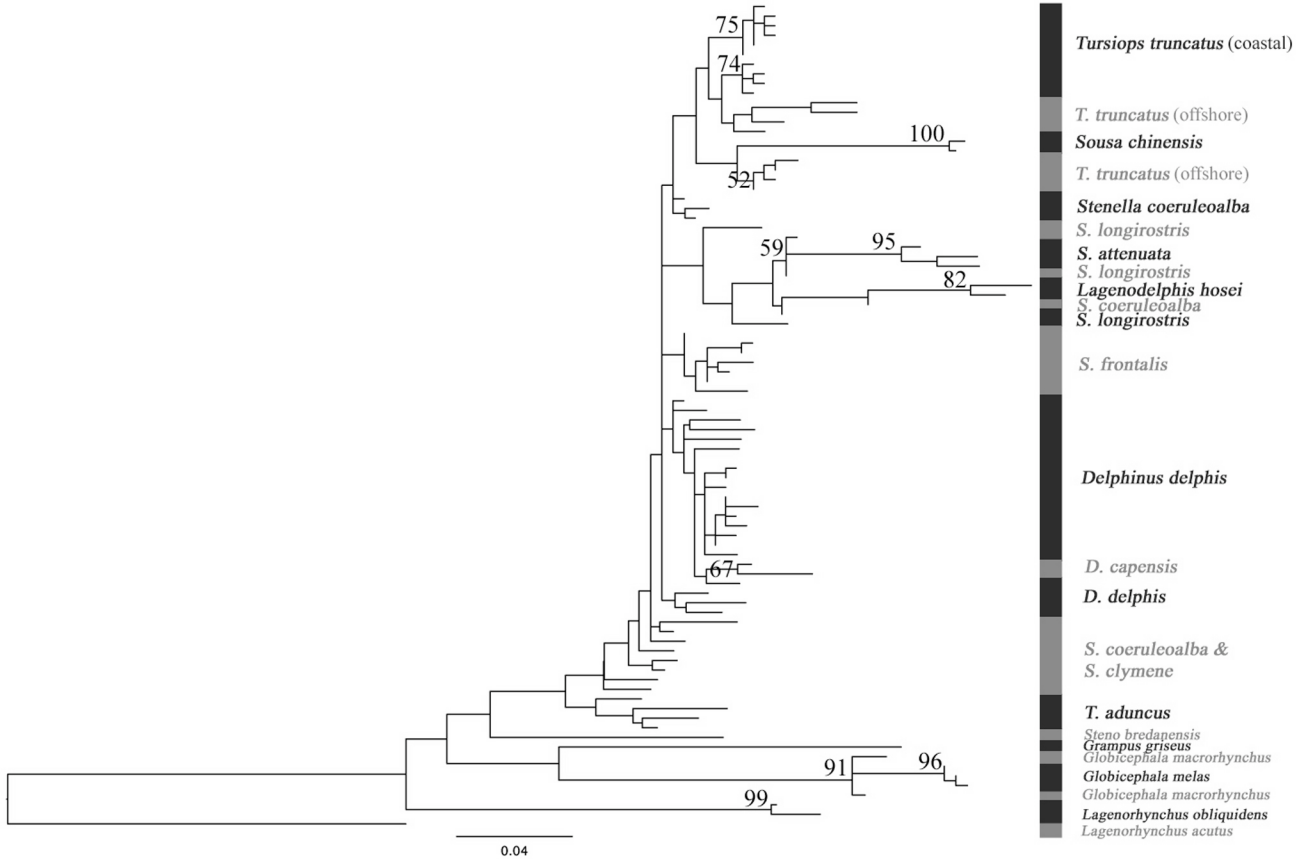

Supplement: Additional file 1 — Phylogenetic analysis of truncated dataset. Phylogenetic tree (best tree) inferred from a pruned dataset of mtDNA control region haplotypes (86 taxa) using the maximum likelihood tool GARLI. Tree is rooted with the outgroup Lagenorhynchus acutus. ML bootstrap values (100 replicates) above 50% are denoted above supported nodes. [file 1471-2148-9-245-S1.PDF]
